# A Comparative Analysis of UAV Photogrammetric Software Performance for Forest 3D Modeling: A Case Study Using AgiSoft Photoscan, PIX4DMapper, and DJI Terra

**DOI:** 10.3390/s24010286

**Published:** 2024-01-03

**Authors:** Sina Jarahizadeh, Bahram Salehi

**Affiliations:** State University of New York, College of Environmental Science and Forestry (SUNY ESF), Department of Environmental Resources Engineering, 1 Forestry Dr., Syracuse, NY 13210, USA; bsalehi@esf.edu

**Keywords:** UAV, photogrammetry, DSM, forest, AgiSoft, PIX4DMapper, DJI Terra

## Abstract

Three-dimensional (3D) modeling of trees has many applications in various areas, such as forest and urban planning, forest health monitoring, and carbon sequestration, to name a few. Unmanned Aerial Vehicle (UAV) photogrammetry has recently emerged as a low cost, rapid, and accurate method for 3D modeling of urban and forest trees replacing the costly traditional methods such as plot measurements and surveying. There are numerous commercial and open-source software programs available, each processing UAV data differently to generate forest 3D modeling and photogrammetric products, including point clouds, Digital Surface Models (DSMs), Canopy Height Models (CHMs), and orthophotos in forest areas. The objective of this study is to compare the three widely-used commercial software packages, namely, AgiSoft Photoscan (Metashape) V 1.7.3, PIX4DMapper (Pix4D) V 4.4.12, and DJI Terra V 3.7.6 for processing UAV data over forest areas from three perspectives: point cloud density and reconstruction quality, computational time, DSM assessment for height accuracy (z) and ability of tree detection on DSM. Three datasets, captured by UAVs on the same day at three different flight altitudes, were used in this study. The first, second, and third datasets were collected at altitudes of 60 m, 100 m, and 120 m, respectively over a forested area in Tully, New York. While the first and third datasets were taken horizontally, the second dataset was taken 20 degrees off-nadir to investigate the impact of oblique images. Results show that Pix4D and AgiSoft generate 2.5 times denser point clouds than DJI Terra. However, reconstruction quality evaluation using the Iterative Closest Point method (ICP) shows DJI Terra has fewer gaps in the point cloud and performed better than AgiSoft and Pix4D in generating a point cloud of trees, power lines and poles despite producing a fewer number of points. In other words, the outperformance in key points detection and an improved matching algorithm are key factors in generating improved final products. The computational time comparison demonstrates that the processing time for AgiSoft and DJI Terra is roughly half that of Pix4D. Furthermore, DSM elevation profiles demonstrate that the estimated height variations between the three software range from 0.5 m to 2.5 m. DJI Terra’s estimated heights are generally greater than those of AgiSoft and Pix4D. Furthermore, DJI Terra outperforms AgiSoft and Pix4D for modeling the height contour of trees, buildings, and power lines and poles, followed by AgiSoft and Pix4D. Finally, in terms of the ability of tree detection, DJI Terra outperforms AgiSoft and Pix4D in generating a comprehensive DSM as a result of fewer gaps in the point cloud. Consequently, it stands out as the preferred choice for tree detection applications. The results of this paper can help 3D model users to have confidence in the reliability of the generated 3D models by comprehending the accuracy of the employed software.

## 1. Introduction

Three-dimensional (3D) information technologies and the evolution of digital data acquisition have recently caught the attention of researchers [1,2]. In order to eliminate human errors in the capture of 3D information, researchers are continually working to find an accurate, precise, sustainable solution [3]. The appearance and geometry of an object or scene can be recovered via 3D reconstruction. The most precise and thorough ways to extract the 3D scene and point cloud among the 3D reconstruction techniques now in use are photogrammetry and laser scanning [4]. A laser scanner is an active sensor that transmits pulses to determine distance, generate a 3D point cloud, and estimate coordinates using onboard navigation systems like Global Positioning System (GPS) or Inertial Navigation System (INS). The flight height, platform speed, sensor field of view, and sensor sampling frequency are just a few of the variables that affect laser scanner point density. However, there are certain drawbacks to laser scanning, including challenges when working in indoor environments, operational sensitivity, a requirement for a significant amount of memory storage, longer computation times, and higher costs [5,6]. Photogrammetry and computer vision, in comparison, have been proposed as solutions to existing limitations [2]. Utilizing overlapping photos taken by visual sensors, photogrammetry is a technology that extracts 3D geometrical data and point clouds. Photogrammetry offers several key advantages over laser scanning, including the ability to use video frames as input and the versatility of using digital images captured with various imaging devices, even smartphones. Additionally, it produces 3D point clouds that contain color information that can be densified. Photogrammetry is also known for its automation capabilities, and most importantly, its cost effectiveness [7,8]. On the other hand, Unmanned Aerial Vehicles (UAVs) are increasingly being used for photogrammetric tasks due to their low cost, low flying altitude, real-time data acquisition capabilities, quick, wide-range sensor availability, and capacity to collect geographic data [9,10,11]. The combination of a low-cost platform, navigation system such as GPS system and IMU system, and high-resolution sensors led to this development [12].

Researchers have introduced a variety of techniques and processes to produce the 3D model from UAV optical data. The significant success of UAV photogrammetry can be largely attributed to the development of Multi-View Stereo (MVS) and Structure From Motion (SfM) algorithms in the field of computer vision, coupled with the advancements in UAV photogrammetric processes. The generation of 3D point clouds, 3D models, and high-quality DSMs has now become straightforward, fast, and user friendly, thanks to the progress in the commercial tools [11,13]. There are over 40 different types of photogrammetric software and tools, both open source and commercial for 3D reconstruction. In order to perform 3D photogrammetric reconstruction, all of these programs generally follow a five-step process: (1) feature detection and matching; (2) triangulation; (3) dense point cloud generation; (4) surface/mesh generation; (5) DSM and orthophoto generation [14].

The advantages of UAV photogrammetry extend across diverse applications and fields including land surface reconstruction [15,16], disaster management [17], and infrastructure applications, such as bridges, roads, railways, and tower inspection [18,19,20], engineering [21], archaeology [11], and most importantly, agriculture and forest management [22,23,24]. However, selecting the best and most suitable tools by industry and user experts for a variety of applications has always been difficult, particularly when it comes to forest modeling with its repeated textures and patterns. Accurate, efficient, and up-to-date data on forest characteristics such as tree height, species, and number of trees have been crucial to the success or failure of urban and forest trees 3D modeling. Canopy Height Models (CHMs) are one of the main techniques for evaluating forest attributes derived using the Digital Surface Model (DSM) that can depict the canopy surface, tree height, and density assessment [25,26]. It can be claimed that the accuracy of the DSM directly affects the accuracy of the retrieved forest parameters, and as a result, can determine whether forest 3D modeling is successful or unsuccessful. Therefore, it is crucial to generate DSM as a photogrammetric product over the forested areas using the best technology available.

Few studies have evaluated various photogrammetric tools, even though many have focused on using UAVs to generate 3D models of forests and the potential for doing so. Svenk 2023 used Keystone, SURF, AgiSoft, and MicMac to generate the point cloud and calculate tree parameters for the forest inventory. An evaluation of the Root Mean Square Error (RMSE) of tree parameters showed that Keystone, SURF, MicMac, and AgiSoft exhibited superior performance in their respective comparison [27]. Terrestrial photos obtained from various visual sensors were employed to compare the 3D models generated by AgiSoft V 1.16, Pix4D V 2.0.89, a combination of Visual SFM V 0.5.22 and SURF V 1.2.0.286, and MicMac V 1.0 on vegetated rock. A point cloud comparison was conducted based on visual evaluation and height profiles. The results indicate that AgiSoft and MicMac exhibit better point cloud accuracy, while Pix4D and the combination of Visual SFM and SURF perform less accurately [28]. Another study compared the DSM produced by AgiSoft, Pix4D, and Leica Photogrammetry Suite (LPS) using ground control points. However, LPS is suitable for airborne (i.e., airplane) photogrammetry and is not effective when it applies to images captured by UAV [29]. A comparison is conducted on height profiles and visual assessments between open-source and commercial photogrammetric software. The results reveal that the software performance depends on applications and texture. Although the ranking of the software depends on the application, Remondino states that AgiSoft generates more reliable and appealing results [30].

It is clear that consumers prefer using the well-known commercial software AgiSoft and Pix4D over other photogrammetric tools for a variety of purposes. Additionally, DJI Terra is a brand new software introduced in 2019, exclusively designed to work with DJI platforms and sensors, making it incomparable to other software [31]. However, given the repeating texture of the forest, a better selection among the existing photogrammetric tools needs to be evaluated considering the application. Also, none of the existing literature has specifically focused on forested areas. In this study, we compare the point clouds and DSM generated over the forest region by AgiSoft, Pix4D, and DJI Terra as well as computational time over the forested areas for forest 3D modeling. The results of this study will assist business and user professionals in identifying constraints and choosing AgiSoft [32], Pix4D [33], or DJI Terra [34] software as the most suitable solution for their project. They will also boost their confidence in their ability to make the right choice instead of investing in expensive projects.

## 2. Methodology and Data Acquisition

**Figure 1:**
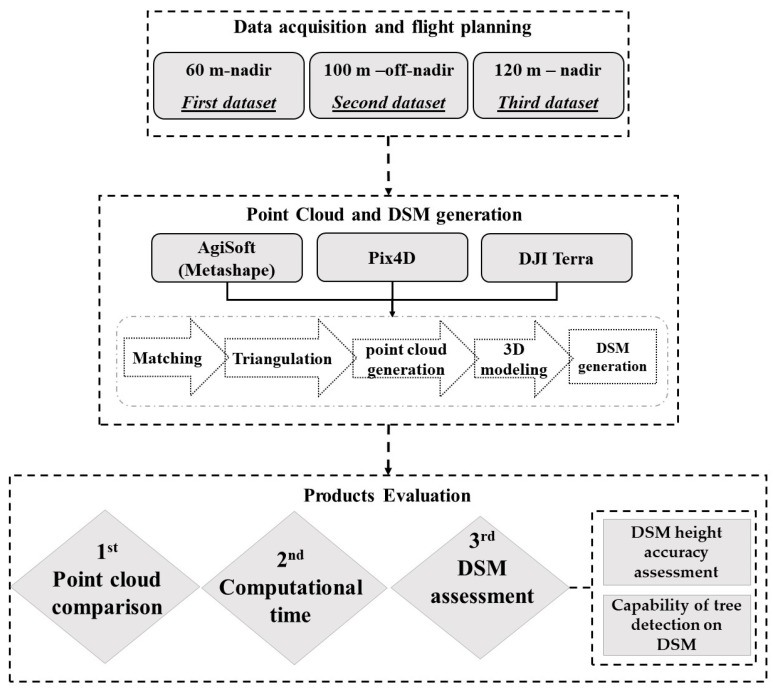
Flowchart of the software comparison strategy in summary.

**Figure 2:**
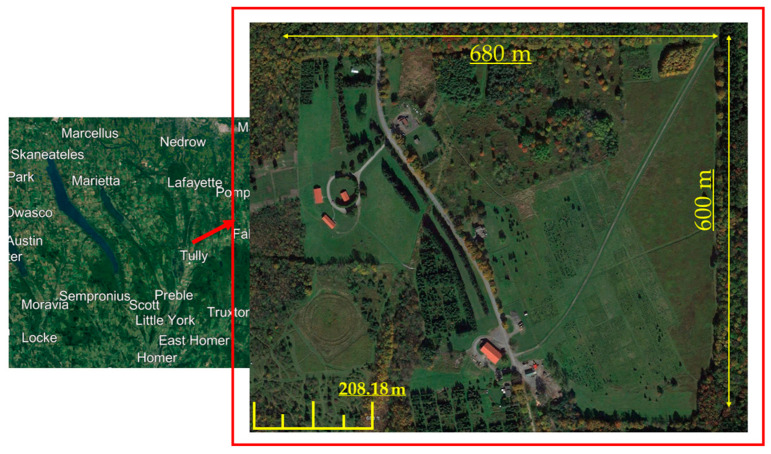
Study area.

The methodology compares the generated dense point cloud and DSM by AgiSoft V 1.7.3 (AgiSoft LLC, St. Petersburg, Russia) [32], Pix4DMapper V 4.4.12 (Pix4D SA, Lausanne, Switzerland) [33], and DJI Terra V 3.7.6 (DJI, Shenzhen, Guangdong, China) [34] as well as their computational time over forested areas. Figure 1 shows a flowchart of the steps that we conduct in this paper. The main steps are (a) data acquisition, (b) product generation, and (c) product evaluation. In the first step, to compare the program under leaf-on situation, three flights using a 20-megapixel optical sensor with 5472 × 3648 resolution and 13.2 × 8.8 mm sensor size were conducted over a section of SUNY ESF Heiberg Forest in Tully, New York about 40 hectares (600 m × 680 m) in total. This area comprises clearcuts, isolated trees, roads, isolated structures, and electricity lines (Figure 2). The first, second, and third flights were conducted at altitudes of 60 m, 100 m, and 120 m, respectively with about 70 to 80 percent overlaps using Site Scan auto pilot application [35]. The first and third datasets were taken horizontally, while the second dataset was taken 20 degrees off-nadir to investigate the impact of oblique images. Table 1 contains a summary of the flight parameters and dataset. The image position and orientation are also provided from the on-board Global Positioning System (GPS) and Inertial Measurement Unit (IMU).

In the next step, AgiSoft Metashape Professional V 1.7.3 [32], PIX4DMapper V 4.4.12 [33], and DJI Terra V 3.7.6 [34] are used for 3D forest modeling. The common workflow of any photogrammetric software for 3D reconstruction and product generation includes feature recognition, matching, triangulation (pose estimation), sparse point cloud generation, point cloud densification, 3D modeling, and DSM generation. While each of these procedures may have distinct names across various software platforms, they must be executed in their respective sequences. While commercial software employs specific equations, it typically uses common algorithms such as a variant of the Scale-Invariant Feature Transform (SIFT) [36] for feature recognition and matching. Additionally, Collinearity conditions (Equation (1)) or Coplanarity conditions are applied in photogrammetry, while the Essential Matrix or Fundamental Matrix is used in computer vision for pose estimation and point cloud generation [37]. For example, the collinearity condition expresses the basic relationship in which an object point and its image point lie on a straight line passing through the sensor perspective center (Equation (1)) [37]. Equation (1) is as follows, where:

- ***R*** is the rotation matrix, *k* is the scale factor, ***a*** is the vector in the object coordinate system, and ***a*′** is the corresponding vector in the sensor coordinate system.
- *X*, *Y*, *Z* are the coordinates of the object point and *X_C_*, *Y_C_*, *Z_C_* are the coordinates of the perspective center (sensor center).
- *c* is the principal distance of the sensor (focal length), $x^{\prime }{}_{0}$ and $y^{\prime }{}_{0}$ are the coordinates of the principal point, and $x^{\prime }$ and $y^{\prime }$ are the corresponding coordinates.

(1) $$\left(\begin{array}{c} x^{\prime }-{x^{\prime }}_{0}\\ y^{\prime }-{y^{\prime }}_{0}\\ -c \end{array}\right)=kR\left(\begin{array}{c} X-X_{C}\\ Y-Y_{C}\\ Z-Z_{C} \end{array}\right)\;\mathrm{or}\;a^{\prime }=kRa$$

Sparse point clouds, dense point clouds, and DSMs are generated using the recommended parameters. Table 2 contains a list of all used preconfigured software settings for AgiSoft, Pix4D, and DJI Terra. All three datasets have been processed on an Intel i9 core CPU laptop processor unit with NVIDIA GeForce GTX 1650 Ti graphic processing units and 64 gigabytes of random-access memory. Finally, the generated point cloud, DSM, and computational time of the listed software are evaluated both independently and in relation to each other, paying particular attention to forest modeling.

## 3. Experiments and Results

The software’s performance assessments focused on comparing three main criteria: (a) point cloud density and reconstruction quality, (b) computational time, and (c) DSM assessment for height accuracy (z) and ability of tree detection on the DSM. 

### 3.1. Point Cloud Density and Reconstruction Quality

**Figure 3:**
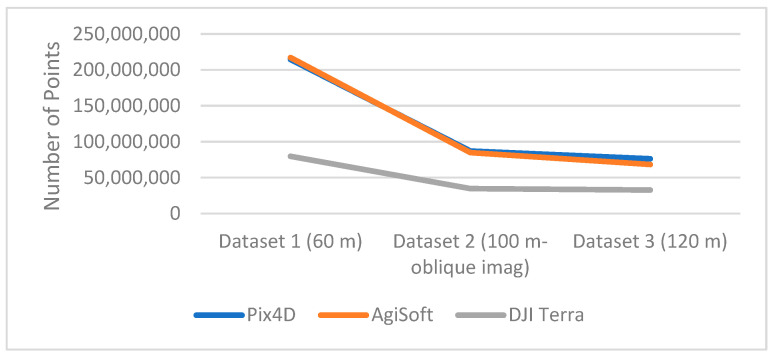
Number of generated points in the dense point cloud.

**Figure 4:**
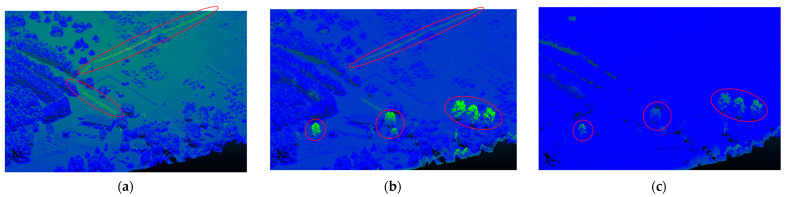
Computed C2C distance between the generated point cloud by (**a**) AgiSoft and DJI Terra, (**b**) Pix4D and DJI Terra, and (**c**) Pix4D and AgiSoft (red circles show the differences).

The performance of dense point cloud generation is evaluated independently by assessing the number of generated points, and by comparing the software’s generated points. Figure 3 compares the point cloud density per dataset for the three software. In all three datasets, Pix4D and AgiSoft produced point clouds that were roughly 2.5 times denser than those produced by DJI Terra. Moreover, Pix4D generates slightly denser point clouds than AgiSoft. The overall generated 3D point cloud quality over various land cover types such as buildings, hills, and trees have shown that there is no significant difference in spatial errors for point clouds of all software. However, due to the different error sources in matching process and repetitive texture in forested areas, there are some gaps created by Pix4D and AgiSoft that can state that the quality of 3D reconstruction is impacted. The software’s generated point cloud can be evaluated for correctness, inaccuracy, and mistake by comparing it to ground truth data. However, distance comparison techniques like the Iterative Closest Point method (ICP) and Multiscale model-to-model Cloud comparison (M3C2) can be used to compare the uniformity, density, and geometry of the point cloud created by various software [38,39,40]. Using the cloud-to-cloud (C2C) distance toolkit in CloudCompare [41] software, which is based on the Iterative Closest Point method (ICP), we have evaluated the overall quality of the generated 3D point cloud over numerous features, such as trees, power lines, buildings, roads, and grass, relatively. On Dataset 2 (oblique images), all software performed nearly identically in terms of completeness (i.e., successfulness in matching process and consequently generated the points for all the existing objects such as trees and buildings). Comparing the other two datasets (Datasets 1 and 3) shows that the DJI Terra generated fewer gaps on forested regions and power lines than Pix4D and AgiSoft, despite producing a fewer number of overall points. In other words, there are some trees and power lines that Pix4D and AgiSoft did not generate any points for (shown by red circles in Figure 4). This indicates that the increased number of points does not necessarily translate into fewer gaps in the point cloud, as DJI Terra utilizes a better key point recognition and matching algorithm. Additionally, in a study, it has been demonstrated that Pix4D generated significant gaps in vegetation regions than AgiSoft which supports our results [42]

### 3.2. Computational Time

**Figure 5:**
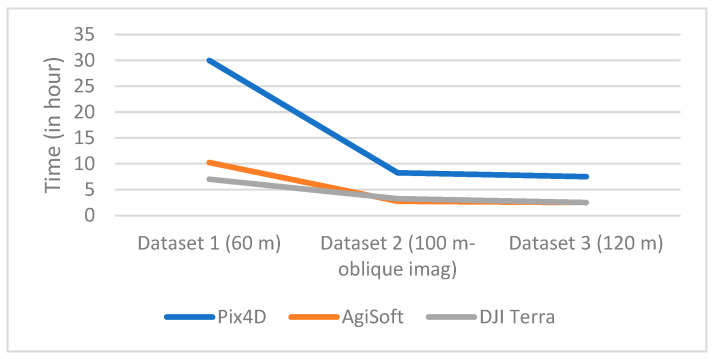
Computational time.

In our evaluation of point cloud density, Pix4D and AgiSoft generated approximately 2.5 times denser point cloud compared to DJI Terra. Consequently, longer computational times for AgiSoft and Pix4D are expected in contrast to DJI Terra. Surprisingly, Pix4D demonstrated an unexpected trend, being roughly three times slower than both AgiSoft and DJI Terra for all datasets (Figure 5). This longer processing time indicates a notable disparity in processing efficiency.

### 3.3. DSM Assessment

DSM assessment has been carried out both quantitatively and qualitatively for all software. The quantitative evaluation involved comparing the standard deviation (SD) and root mean square error (RMSE). The SD and RMS are calculated using height differences between AgiSoft, Pix4D, and DJI Terra from elevation profiles derived from DSMs of various land cover types including single trees, patches of trees, buildings, and roads. A lower RMSE means a better match between generated elevations by two software. On the other hand, the SD gives a measure of how much the elevations deviate from their mean. A significant difference indicates a systematic error. Subsequently, we assessed the DSM quality for tree detection applications using DSM.

#### 3.3.1. DSM Height Accuracy Assessment Using Elevation Profile

**Figure 6:**
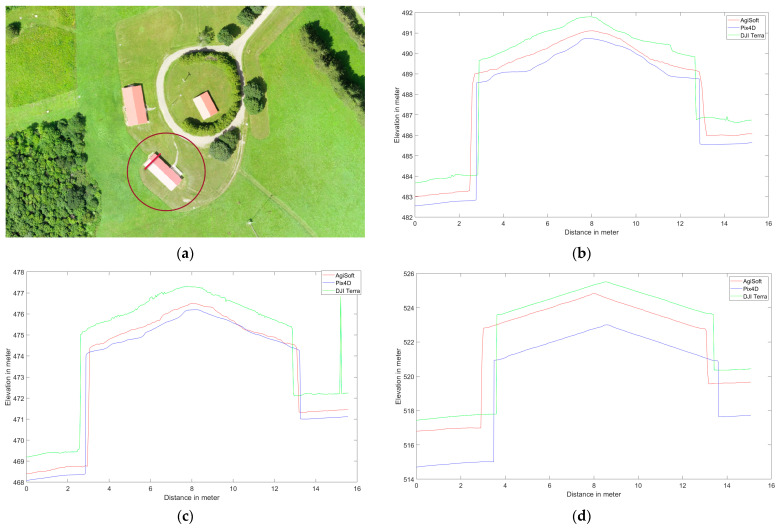
Elevation profile from Pix4D (blue), AgiSoft (red), and DJI Terra (green) on a building, (**a**) profile line (red circle shows the picked feature), (**b**) Dataset 1 (60 m), (**c**) Dataset 2 (100 m oblique images), and (**d**) Dataset 3 (120 m).

**Figure 7:**
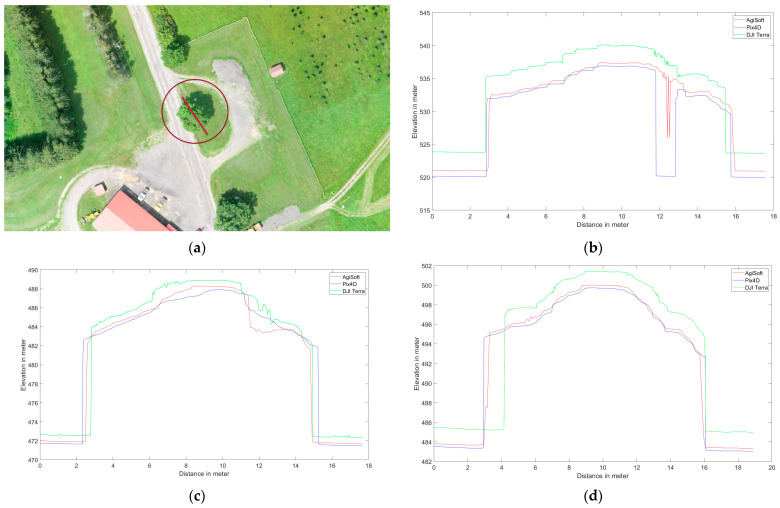
Elevation profile from Pix4D (blue), AgiSoft (red), and DJI Terra (green) on a tree, (**a**) profile line(red circle shows the picked feature), (**b**) Dataset 1 (60 m), (**c**) Dataset 2 (100 m oblique images), and (**d**) Dataset 3 (120 m).

**Figure 8:**
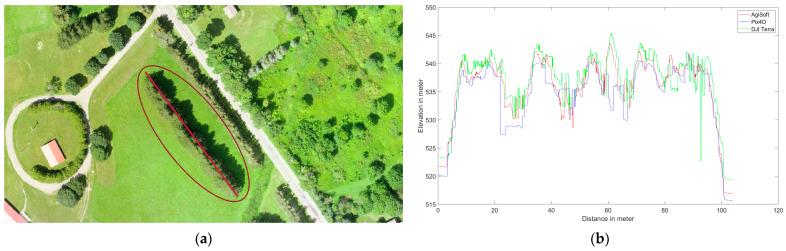

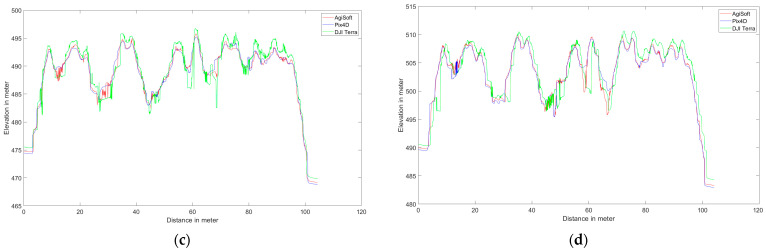
Elevation profile from Pix4D (blue), AgiSoft (red), and DJI Terra (green) on a patch of the trees, (**a**) profile line (red circle shows the picked feature), (**b**) Dataset 1 (60 m), (**c**) Dataset 2 (100 m oblique images), and (**d**) Dataset 3 (120 m).

**Figure 9:**
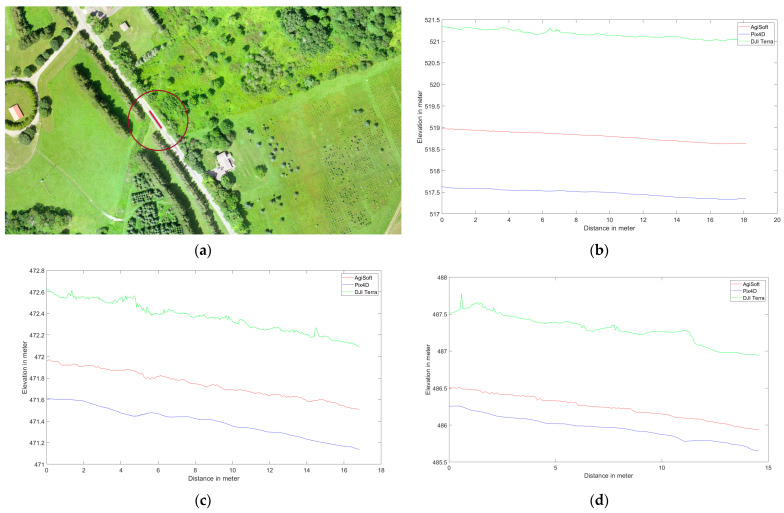
Elevation profile from Pix4D (blue), AgiSoft (red), and DJI Terra (green) on a road, (**a**) profile line (red circle shows the picked feature), (**b**) Dataset 1 (60 m), (**c**) Dataset 2 (100 m oblique images), and (**d**) Dataset 3 (120 m).

Several elevation profile examples are retrieved for various land covers including buildings (Figure 6), trees (Figure 7), tree patches (Figure 8), and roads (Figure 9) to quantitatively evaluate the generated DSMs. Elevation profiles showed consistent vertical shifts among the generated DSMs for various land cover types and datasets. Specifically, the elevation profile extracted from DJI Terra’s DSM consistently is higher than AgiSoft, whereas Pix4D consistently has a lower elevation compared to AgiSoft and DJI Terra. The elevation differences between AgiSoft and DJI Terra are up to 2.5 m for the first dataset, 0.9 m for the second dataset, and 1.5 m for the third dataset. In contrast, the elevation differences between Pix4D and AgiSoft are up to 1 m for the first dataset and 0.5 m for the second and third datasets. It shows that the 3D elevation from Pix4D AgiSoft is distinct from the DJI Terra result while also being similar to each other. The number of generated points may be the root cause of the significant elevation differences between DJI Terra and two other software. Fewer points within a pixel can lead to distinct elevations in the DSM, given that the elevation of each pixel is computed as the weighted total of its internal points. Furthermore, vertical shifts between the generated DSMs may be impacted by the points distribution. The various closed sophisticated algorithms that are applied in commercial software are another potential cause of vertical shifts. In general, when features are found at a higher elevation section of the research area (i.e., on top of a hill), the amount of the vertical shift is reduced since the features are closer to the drone, and thus have a lower flying height than in other areas.

The utilization of oblique images rather than vertical ones reduces the vertical shifts across all software. The greater intersection angles in oblique images enhance the accuracy of elevation estimation through improved collinearity equations [43]. The third dataset displays fewer vertical shifts than the first dataset, a reason that may be attributed to a higher flight altitude. Generally, higher flight altitudes often result in lower spatial resolution and consequently reduced detail and repetitive textures, especially in areas with dense forest cover, where repetitive textures can affect the accuracy of matching and elevation data. In the analysis of the first and second datasets, elevation spikes can be seen on trees in Pix4D and AgiSoft. All applications and datasets also exhibit slight horizontal shifts. Although there are horizontal and vertical shifts, the Pix4D and AgiSoft images are more pleasing and smoother for flat surfaces like roadways than DJI Terra.

**Figure 10:**
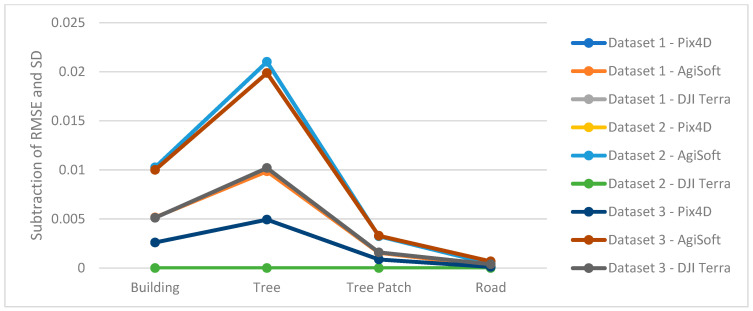
Difference between RMSE and SD.

It can be said that the results from DJI Terra are more compelling, especially when applied to natural features such as trees. It is common to see numerous slight height discrepancies in areas covered with vegetation, such as dense trees. However, Pix4D and AgiSoft do not appear to have as many details extracted as DJI Terra which suggests a potential advantage to capture finer details in vegetated areas. The accuracy and adaptability across various datasets are measured by the root mean square error (RMSE) metric and the standard deviation (SD) calculated for height differences between AgiSoft, Pix4D, and DJI Terra. Utilizing the standard deviation (SD) metric defines a range that encompasses the average to identify outliers. It can be concluded that the distribution of errors is normal and there are no systematic errors or outliers in the outputs if the RMSE and standard deviation (SD) values are similar [44,45]. The small discrepancies between RMSE and SD confirm the absence of systematic inaccuracy (bias) among the DSMs produced by all software (Figure 10). Furthermore, it shows how close the 3D profile models from Pix4D, AgiSoft, and DJI Terra are to one another.

#### 3.3.2. Capability of Tree Detection on DSM

**Figure 11:**
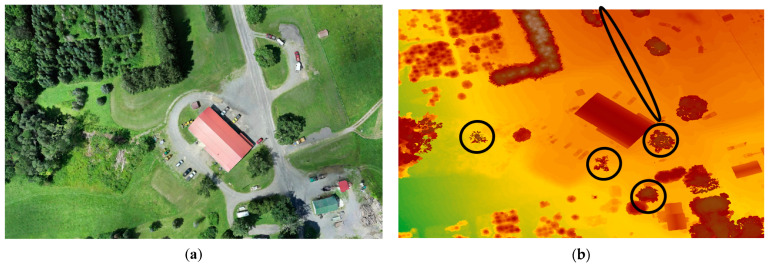

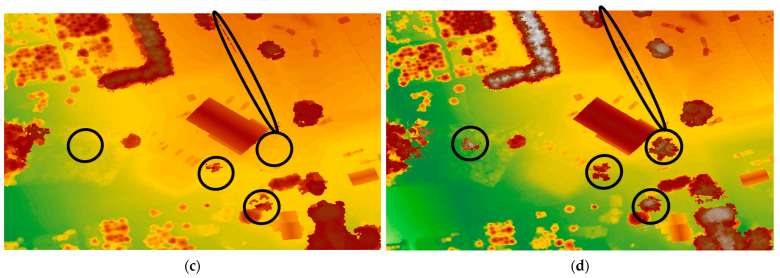
(**a**) Orthophoto, generated DSM on Dataset 1 (60 m) by (**b**) AgiSoft, (**c**) Pix4D, and (**d**) DJI Terra, black circles indicate the differences.

**Figure 12:**
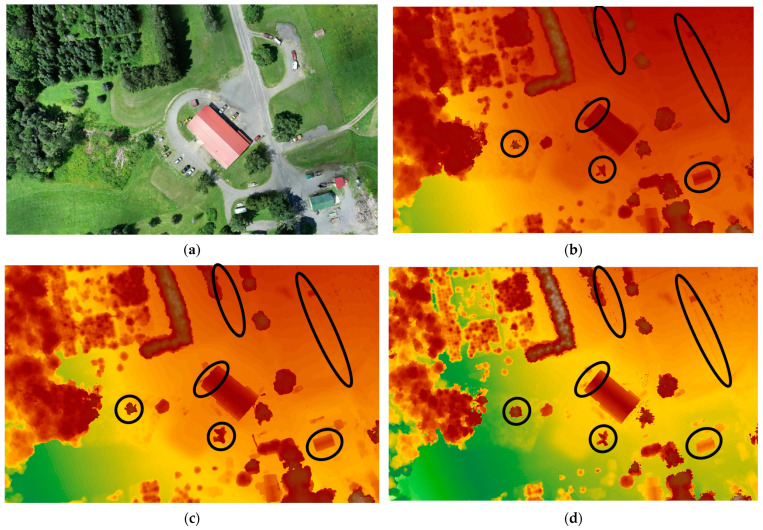
(**a**) Orthophoto, generated DSM on Dataset 2 (100 m oblique images) by (**b**) AgiSoft, (**c**) Pix4D, and (**d**) DJI Terra, black circles indicate the differences.

**Figure 13:**
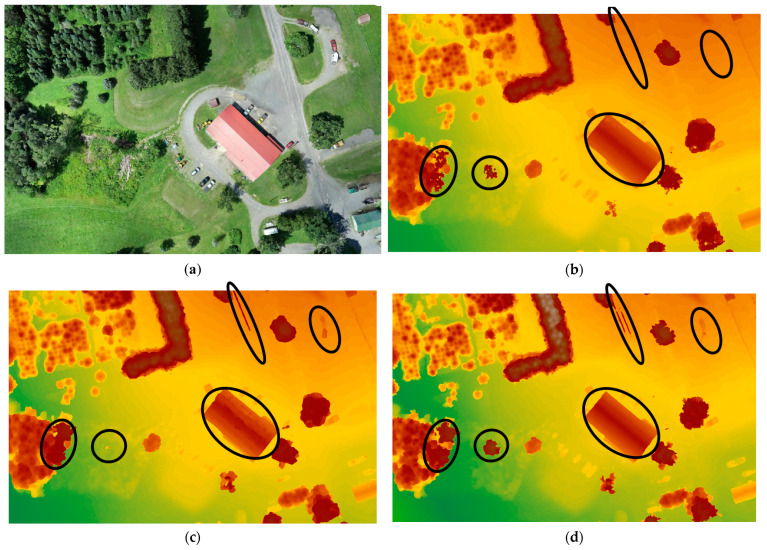
(**a**) Orthophoto, generated DSM on Dataset 3 (120 m) by (**b**) AgiSoft, (**c**) Pix4D, and (**d**) DJI Terra, black circles indicate the differences.

The evaluation of tree detection capabilities in the generated DSMs has been carried out through visual comparisons. The DSMs were generated using Pix4D, AgiSoft, and DJI Terra and were visually assessed for their effectiveness in accurately detecting trees. The results show an obvious elimination of some trees (i.e., missing some trees) in the DSMs generated by Pix4D and AgiSoft which can raise considerations regarding the completeness and accuracy of tree detection in these software outputs. Despite generating around 2.5 times fewer points than Pix4D and AgiSoft, DJI Terra was still able to generate and detect a more detailed DSM, resulting in the identification of several trees that were not present in the DSMs generated by Pix4D and AgiSoft. Examples of missing trees are highlighted with black circles in Figure 11, representing Dataset 1 (60 m), Figure 12 for Dataset 2 (100 m oblique images), and Figure 13 for Dataset 3 (120 m). Furthermore, DJI Terra’s DSM is smoother than that generated by Pix4D and AgiSoft. The possible causes include (1) the use of a better outlier rejection approach in the DJI Terra that causes the generation of a better DSM [46], and (2) the improved point distribution achieved by DJI Terra. Furthermore, DJI Terra and AgiSoft demonstrated superior precision in capturing the corners and edges of buildings compared to Pix4D. In general, it can be said that the DJI Terra outperforms Pix4D and AgiSoft in forestry areas by spotting more single trees and identifying the edge of the single trees within tree patches.

## 4. Conclusions

This study was conducted to assist industry and professional users in discovering and choosing the best software among AgiSoft, Pix4D, and DJI Terra for forest 3D modeling purposes as well as to boost their confidence in making the right choice instead of investing in expensive projects. Three flights within altitudes of 60, 100, and 120 m were conducted to evaluate the point cloud density and reconstruction quality, computational time, and DSMs for height accuracy (z) and ability of tree detection both quantitively and qualitatively over the forested area. The results show that Pix4D and AgiSoft generated denser point clouds than DJI Terra. However, DJI Terra provided a better point cloud of trees than the other two software, likely due to utilizing an enhanced matching algorithm. As a result, DJI Terra generated an accurate DSM with fewer gaps than AgiSoft and Pix4D. Despite the vertical shift in height values on generated DSM, DJI Terra performed better in terms of modeling trees and building shapes. However, AgiSoft and Pix4D performed better in generating the road elevation profile than the DJI Terra. In general, Pix4D generated the highest elevation, followed by AgiSoft, and lastly DJI Terra. Finally, the computational time comparison reveals that the processing time of AgiSoft and DJI Terra is roughly half that of Pix4D. Future research can contribute to enhancing our understanding by evaluating the accuracy of each product against referenced ground truth data and comparing them to other commercial software as we only relatively evaluated AgiSoft, Pix4D, and DJI Terra.

## Figures and Tables

**Table 1:** Datasets and flight parameters.

Platform	Flight Height	Front Overlap	Side Overlap	Gimbal Angle	Resolution	Number of Images	Condition
**Dataset 1** **(First Flight)**	~60 m	70	80	90 degrees	GSD ~1.98 cm	1829	leaf on
**Dataset 2** **(Second Flight)**	~100 m	70	65	70 degrees	GSD ~4.60 cm	768	leaf on
**Dataset 3** **(Third Flight)**	~120 m	70	65	90 degrees	GSD ~3.99 cm	704	leaf on

**Table 2:** Photogrammetric tools processing setting.

	Sparse Point Cloud	Dense Point Cloud	DSM
**AgiSoft**	High (Full image size)	Medium (down sampled image by factor 2)	High
**Pix4D**	Full (Full image size)	Multiscale with half image size (down sampled image by factor 2)	Automatic
**DJI Terra**	High (Full image size)	Height	High

## Data Availability

Data are contained within the article.
